# Comparative evaluation of biofilm-forming capacity in uropathogenic and commensal *Escherichia coli*

**DOI:** 10.3389/fcimb.2025.1570422

**Published:** 2025-07-31

**Authors:** Rashmi P. Mahale, Anuradha K, Adeline Princy, Yogeesh D. Maheshwarappa, Mahadevaiah Neelambike Sumana

**Affiliations:** ^1^ Department of Microbiology, JSS Medical College and Hospital, JSS Academy of Higher Education and Research, Mysuru, India; ^2^ Department of Microbiology, Mysore Medical College and Research Institute, Mysuru, India; ^3^ Quorum Sensing Laboratory, School of Chemical and Biotechnology, Sastra Deemed to be University, Thanjavur, India

**Keywords:** biofilm production, uropathogenic *Escherichia coli*, multiplex PCR, urinary tract infection, Congo-red method, tissue culture plate method

## Abstract

**Introduction:**

*Escherichia coli* (*E. coli*) causes most cases of the urinary tract infections (UTIs) via virulence factors like biofilms. This study identifies key phenotypic and genotypic virulence attributes of Uropathogenic Escherichia coli.

**Methodology:**

A total of 180 uropathogenic *E. coli* (UPEC) isolated from patients with different categories (cystitis, pyelonephritis, recurrent UTI, catheter-associated UTI, and asymptomatic bacteriuria) of UTI and 30 commensal *E. coli* isolated from healthy individuals were evaluated for biofilm production by phenotypic methods using tissue culture plate, tube adherence, and Congo red method, and RT-PCR was used to genetically characterize them.

**Results:**

This study analyzed 1,600 urine samples from UTI patients, with 498 showing significant bacterial growth and 180 identifying *E. coli* as the pathogen. The female-to-male ratio of UTI cases was 0.74. Antibiotic susceptibility testing revealed 100% sensitivity to tigecycline and fosfomycin as well as 89.44%, 86.11%, 81.66%, and 72.22% sensitivity to nitrofurantoin, amikacin, imipenem, and meropenem, respectively. Only 64.44% were sensitive to ciprofloxacin, with 10% being multidrug-resistant (MDR). Moreover, 18.33% of the UPEC isolates produced mettalo-beta-lactamases (MBL), and 13.33% produced *AmpC* beta-lactamases. Biofilm production was observed in 72.22% of UPEC isolates compared to 16.66% in commensal isolates. The biofilm-forming UPEC, compared to commensal *E. coli*, has significantly higher antibiotic resistance, with a 128-fold reduction in ciprofloxacin susceptibility. Additionally, the *fimH* gene was detected in 98.33% of the UPEC isolates.

**Conclusion:**

This study shows that UPEC strains produce specific virulence determinants like adhesion to uroepithelial cells. Screening for virulence factors should be integrated into microbiology laboratories. Specific virulence genes linked to UPEC may serve as potential targets for prophylactic strategies to prevent recurrent infections and improve management.

## 1 Introduction

Urinary tract infection (UTI) results in the inflammation of the urinary tract due to the growth of a significant number (>10^5^ CFU/ml) of uropathogens. UTIs can be classified depending on the severity, such as urosepsis, pyelonephritis, and cystitis. Clinically, UTIs can be classified as uncomplicated and complicated UTIs. UTI is the most common reason for visiting a healthcare facility, with approximately 150 million people developing UTIs annually. Women are more prone to develop UTI, and it is estimated that 40% of women develop UTI at least once during their lifetime, and about 11% of women above the age of 18 years develop an episode of UTI per year. Though UTIs can be treated effectively by antibiotics, recurrence is widespread. Recurrent UTIs may be due to bacterial virulence factors and host deficiencies (Foxman and Brown, 2003; Micali et al., 2014; Flores-Mireles et al., 2015).

Uropathogenic *Escherichia coli* (UPEC) is responsible for approximately 90% of community-acquired and 50% of nosocomial UTIs. This may be due to a multitude of virulence factors (VFs), which facilitate them to survive, grow, and persist in the adverse settings of the urinary tract (Epp and Larochelle, 2010; Soto et al., 2011; Jhang and Kuo, 2017). These virulence factors are coded by large regions of mobile genomic material called genomic islands (GI). The genomic islands containing more than one virulence gene are called pathogenicity islands (PAIs). VFs in UPEC can be classified as cell surface factors, including type 1 fimbriae (*fimH*), P fimbriae (*pap*), S fimbriae (*sfa*), F1C fimbriae, afimbrial adhesion I (*afaI*), thin aggregative fimbriae (also called curli), flagella, capsule, outer membrane proteins, and lipopolysaccharides. Additionally, there are exported virulence factors, which include alpha-haemolysin (*hlyA*), cytotoxic necrotizing factor 1, cytolethal distending toxin, secreted autotransporter toxin, cytolysin A, and siderophores like enterobactin, aerobactin (aer), and yersiniabactin (Hooton and Stamm, 1997; Oelschlaeger et al., 2002; Vila et al., 2002; Yan and Polk, 2004; Wiles et al., 2008).

The cell surface factors enable UPEC to form multicellular communities called biofilms. Biofilm formation allows UPEC to persist in the urinary tract by providing several survival advantages, including antibiotic resistance, expression of various virulence factors through quorum sensing, and resistance to host defense mechanisms such as phagocytosis. Infections caused by biofilm-producing UPEC strains are challenging to treat due to their high levels of resistance to antibiotics (Mobley and Warren, 1996; Hooton and Stamm, 1997; Oelschlaeger et al., 2002; Wiles et al., 2008). These infections have been linked to recurrent infections and prolonged hospital admissions, resulting in increased healthcare costs and a greater risk of acquiring additional nosocomial infections. Consequently, there is an elevated likelihood of increased morbidity and mortality. Therefore, it is crucial to determine the phenotypic and genotypic virulence attributes of UPEC, especially its biofilm-producing capability. This approach aids in improving the management and prognosis of UTIs, reducing their economic burden, enhancing treatment plans, assessing patient risks, improving infection control procedures, and allocating resources to reduce antimicrobial resistance. Thus, the present study aimed to determine the biofilm-forming capacity of UPEC.

## 2 Methodology

### 2.1 Sample collection/study design

This laboratory-based prospective study was conducted at a tertiary care hospital in Mysuru, Karnataka. The present study evaluated and compared the biofilm-forming capacity of UPEC and intestinal commensal *E. coli.* Urine samples sent for culture and sensitivity from clinically suspected cases of UTI were received in the laboratory and processed according to the standard protocol.

A total of 180 urine samples with significant pyuria (≥5 inflammatory cells) and significant growth of *E. coli* (≥10^5^ log colony-forming units) were included in the study. Gram-positive and Gram-negative organisms other than *E. coli* isolated from the urine specimens, *E. coli* isolated in insignificant numbers (<10^5^), and repeat isolates from the same patient were excluded from the study. Relevant patient demographic and clinical information details were collected from the hospital information system and medical records. Stool samples from healthy adults were collected after informed consent and cultured on MacConkey agar. The lactose-fermenting colonies identified as *E. coli* were included in the study as intestinal commensal *E. coli.* A total of 30 intestinal commensal *E. coli* were included in the study.

### 2.2 Sample processing

*Microscopic examination*: Uncentrifuged urine samples were examined microscopically using the wet mount technique under a 40× objective lens to screen for inflammatory cells, red blood cells, and organisms. A finding of ≥5 inflammatory cells per high-power field (HPF) was considered significant pyuria (Baron and Finegold, 2007).

*Semiquantitative culture*: Semiquantitative culture was carried out using a sterile calibrated nichrome wire loop delivering 0.001 mL of urine (measuring 2 mm in internal diameter) on Urichrome agar (UCA). The inoculated UCA plates were incubated aerobically at 37°C for 18 to 24 h. After incubation, violet-colored *E. coli* colonies on UCA were counted using a magnifying lens. The number of colonies counted was multiplied by 1,000 to calculate the colony-forming unit (CFU) per milliliter of urine. The presence of 100,000 or more colonies was considered a significant bacteriuria. The violet-colored *E. coli* colonies were confirmed using an automated identification system (Vitek-2 compact system by bioMérieux, France).

*Antimicrobial susceptibility testing (AST)*: Antibiotic sensitivity testing was carried out using an automated Vitek-2 compact system by bioMérieux, France. The results were interpreted according to the CLSI guidelines (M-100, 31st edition) (Clinical and Laboratory Standards Institute, 2020). Additionally, the antimicrobial susceptibility testing for fosfomycin was carried out using an Epsilometer strip (E-strip) on cation-adjusted Muller Hinton Agar (MHA) (HiMedia Laboratories, Mumbai, India).

### 2.3 Phenotypic methods for the detection of biofilm formation among UPEC

This study used three phenotypic methods to detect biofilm formation among UPEC and intestinal commensal *E. coli*. Among the three methods, the tissue culture plate method was considered the gold standard for biofilm detection, but it is labor-intensive and technically demanding. Hence, we evaluated the efficacy of simpler alternatives, the Congo red agar method (CRA) and tube adherence methods, to determine their reliability as screening tools.

*Tissue culture plate method (TCM)*: The organisms from fresh agar plates were inoculated in trypticase soy broth with 2% glucose and incubated for 24 h at 37°C in stationary conditions. The broth was diluted 1:100 with fresh medium. Individual sterile polystyrene wells of a 96-well flat-bottomed tissue culture plate (TCP) were filled with 200 µL of diluted cultures. Only the medium in the well served as a control to check the sterility of the media and the nonspecific binding of the media to the well. The TCP was incubated for 24 h at 37°C. After incubation, the contents of each well were gently removed by tapping the plates. The wells were washed four times with 0.2 mL of phosphate-buffered saline (PBS, pH 7.2) to remove free-floating planktonic bacteria. Biofilms formed by adherent “sessile” organisms in a plate were fixed with sodium acetate (2%) and stained with crystal violet (0.1% w/v) for 1 min. Excess stain was rinsed off by washing with deionized water, and the plates were kept for drying. The biofilms formed and stained uniformly with crystal violet. The optical density (OD) of stained adherent bacteria was determined with a micro-ELISA auto reader (model 680, Bio-Rad) at a wavelength of 570 nm (OD570). This OD value was considered as an index of bacterial adherence/biofilm (Christensen et al., 1985).

The experiment was performed in triplicate; the data generated was averaged, and the standard deviation was calculated. To compensate for background absorbance, OD readings from sterile medium, fixation, and dye were averaged and subtracted from all test values. *S. epidermidis* ATCC 35984 was used as a positive control for biofilm formation, and *S. epidermidis* ATCC 12228 was used as a negative control.

Classification of bacterial adherence was based on the OD values obtained for individual strains, categorized as strongly positive, moderately positive, weakly positive, and non-biofilm forming, as indicated in **Table 1** (Park et al., 2009).

**Table 1:** Classification of bacterial adherence.

Mean optical density	Adherence	Biofilm formation
<0.120	Non/weak	None/weak
0.120–0.240	Moderate	Moderate
>0.240	Strong	Strong

*Tube adherence method (TAM)*: A loopful of the test organisms was inoculated into a test tube containing 10 mL of sterile brain heart infusion broth. The tube was incubated aerobically at 36°C ± 1°C for 24 h. The tube content was discarded, and the tube was washed with 9 mL phosphate buffer saline at ph 7.2. The biofilm formed was fixed by adding 10 mL of freshly prepared sodium acetate (2%) into each tube and leaving it for 10 min. The contents of the tube were discarded, and 10 mL of crystal violet (0.1%) was added to each tube and left at room temperature for 30 min. The stain was discarded, and the washing step was repeated. The tubes were allowed to dry in an inverted position at room temperature. Interpretation: Biofilm formation was detected by the presence of visible film on the wall and bottom of the tube. The biofilm was graded visually as absent, moderate, and strong biofilm formation (Christensen et al., 1982).

*Congo red agar method (CRA)*: Congo red agar was prepared by mixing brain heart infusion agar (37 g) and sucrose (50 g) in 800 mL of distilled water and autoclaving the mixture. Congo red stain (200 mL) was added when the agar cooled to 55°C. The test organisms were plated onto the CRA plate and incubated aerobically at 37°C for 24 h. Interpretation: A black-colored colony was considered a biofilm producer (Freeman et al., 1989).

### 2.4 Genotypic attributes of biofilm among *E. coli* isolates

Deoxyribonucleic acid (DNA) was extracted from fresh 24-h cultures of the test isolates grown on nutrient agar using a commercial Spinster total DNA extraction kit (ADT Biotech, Kuala Lumpur, Malaysia) following the manufacturer’s protocol. The extracted nucleic acid was subjected to real-time multiplex polymerase chain reaction (PCR) for the detection of six adhesion genes. Type-it HRM master mix (purchased from Qiagen company from Germany) was used according to the manufacturer’s instructions. Multiplex PCR was employed to identify six adhesion genes, namely, *fimH* (465 bp), *papC* (203 bp), *papGII* (190 bp), *papGIII* (258 bp), *afa/draBC* (593 bp), and *sfa/focDE* (408 bp). The details of the primers are given in **Table 2**. Based on the melting curve of gene amplicons, a multiplex PCR was designed. The temperature profile for real-time PCR is detailed in **Table 3**. The melting curve analysis was done at 70°C to 90°C.

**Table 2:** The details of the primers’ reference (Yamamoto et al., 1995; Johnson and Stell, 2000; Usein et al., 2001; Wang et al., 2002; Mapes et al., 2007; Park et al., 2009; Yun et al., 2014; Munkhdelger et al., 2017; Rashki et al., 2017).

Gene	Primer	Primer sequence 5′ to 3′	Number of base pairs
*fim H*	fim H F	AACAGCGATGATTTCCAGTTTGTGTG	26
	fim H R	ATTGCGTACCAGCATTAGCAATGTCC	26
*pap C*	pap C F	GTGGCAGTATGAGTAATGACCGTTA	25
	pap C R	ATATCCTTTCTGCAGGGATGCAATA	25
*Pap GII*	pap G II F	GGGATGAGCGGGCCTTTGAT	20
	pap G II R	CGGGCCCCCAAGTAACTCG	19
*PapGIII*	pap G III F	GGCCTGCAATGGATTTACCTGG	22
	pap G III R	CCACCAAATGACCATGCCAGAC	22
*afa/draBC*	afa/dra BC F	GGCAGAGGGCCGGCAACAGGC	21
	afa/dra BC R	CCCGTAACGCGCCAGCATCTC	21
*sfa/focDE*	sfa/foc DE F	CTCCGGAGAACTGGGTGCATCTTAC	25
	sfa/foc DE R	CGGAGGAGTAATTACAAACCTGGCA	25

**Table 3:** Temperature profile of multiplex PCR.

Step	Temperature and time	Number of cycles
Denaturation	95°C for 5 min	Hold
Annealing	95°C for 10 s	45
Elongation	55°C for 30 s	45
Extension	72°C for 15 s	45

*Statistical analysis*: Statistical analysis was performed using the Statistical Package for Social Sciences (SPSS) version 17.0 software package. We applied descriptive statistics such as percentage, mean, and standard deviation. Chi-square test was applied. The difference and association were interpreted as statistically significant when *p* was less than 0.05.

## 3 Results

### 3.1 Patients’ demographic and clinical characteristics

In this study, 1,600 urine samples were received from patients with suspected UTIs. Out of these, 918 (57.37%) samples were from female patients and 682 (42.62%) samples were from male patients, with a female-to-male ratio of 0.74. The majority of the patients belonged to the age group of 20–40 years (*n* = 618, 38.62%), followed by 50–60 years (*n* = 266, 16.62%). **Table 4** shows the demographic and clinical profile of the study samples. Of the 1,600 urine samples received, 988 (61.75%) samples were from patients with cystitis, 188 (11.75%) from pyelonephritis, 314 (19.62%) from catheterized patients, 72 (4.5%) from asymptomatic bacteriuria (ABU), and 38 (2.37%) from patients with recurrent UTIs.

**Table 4:** Demographic and clinical profile.

Patient characteristics	Total number	Total in percentage
Gender		
Male	682	42.62
Female	918	57.37
Age (years)		
<10	118	12.85
10–20	36	3.92
20–40	364	39.65
40–50	124	13.50
50–60	151	16.44
60–70	69	7.51
>70	56	6.10
Diagnosis/clinical condition		
Cystitis	988	61.75
ASB	72	4.5
Pyelonephritis	188	11.75
Recurrent UTI	38	2.37
CAUTI	314	19.62

### 3.2 Semiquantitative culture results

Of the 180 UPEC isolates included in the study, 101 (56.11%) were from patients with cystitis, 31 (17.22%) were from patients with pyelonephritis, 28 (15.55%) were from catheterized patients, 11 (6.11%) were from patients with recurrent UTI, and nine (5%) were from patients with asymptomatic bacteriuria.

### 3.3 Antibiotic susceptibility pattern of UPEC and commensal *Escherichia coli*

As given in **Table 5**, the antibiotic susceptibility profile for UPEC isolates in this study revealed high sensitivity to nitrofurantoin (89.44%), followed by amikacin (86.11%), imipenem (72.77%), meropenem (71.66%), and cefoparazone/sulbactam (56.11%). Notably, all UPEC isolates (100%) were sensitive to fosfomycin and tigecycline. None of the commensal *E. coli* were multidrug-resistant; however, three isolates showed resistance to both carbapenems tested. None of the commensal isolates were MBL or AmpC producers. Antimicrobial resistance was compared between UPEC and fecal *E. coli* isolates. The UPEC isolates showed a higher degree of resistance when compared to the fecal isolates.

**Table 5:** Antibiotic susceptibility pattern of UPEC and commensal *Escherichia coli*.

Antimicrobial agent	UPEC (180)		Commensal *E. coli* (30)		Chi-square	*P*-value
	Sensitivity	Resistance	Sensitivity	Resistance		
Fosfomycin (E- test)	180 (100%)	0	30 (100%)	–	NA	NA
Tigecycline	180 (100%)	0	30 (100%)	–	NA	NA
Nitrofurantoin	161 (89.44%)	19 (10.55%)	26 (86.66%)	4 (13.33%)	47.33	0.001*
Amikacin	155 (86.11%)	25 (13.88%)	29 (96.66%)	1 (3.33%)	2.64	0.104
Gentamycin	143 (79.44%)	37 (20.55%)	21 (70%)	9 (30%)	1.34	0.247
Imipenem	131 (72.77%)	49 (27.22%)	28 (93.33%)	2 (6.66%)	5.91	0.015
Meropenem	129 (71.66%)	51 (28.33%)	26 (86.66%)	4 (13.33%)	2.99	0.084
Ciprofloxacin	116 (64.44%)	64 (35.55%)	23 (76.66%)	7 (23.33%)	1.72	0.190
Cefaperazone/sulbactam	101 (56.11%)	79 (43.88%)	26 (86.66%)	4 (13.33%)	10.04	0.001*
Nalidixic acid	92 (51.11%)	88 (48.88%)	21 (70%)	9 (30%)	3.69	0.055
Cefepime	59 (32.77%)	121 (67.22%)	24 (80%)	6 (20%)	23.99	0.001*
Ceftriaxone	49 (27.22%)	131 (72.77%)	18 (60%)	12 (40%)	12.72	0.001*
Cefuroxime	44 (24.44%)	136 (75.55%)	15 (50%)	15 (50%)	8.31	0.003*
Amoxiclav	32 (17.77%)	148 (82.22%)	20 (66.66%)	10 (33.33%)	32.99	0.001*
Ampicillin	14 (7.77%)	166 (92.22%)	17 (56.66%)	13 (43.33%)	54.29	0.001*

E- test, Epsilometer test, Significant P value ≤ 0.05. NA, not applicable.* Significant association.

Among the 180 UPEC isolates, 18 were identified as multidrug-resistant (MDR), showing resistance to at least one agent in three or more antibiotic classes. As given in **Table 6**, among the 18 MDR isolates, seven (38.88%) isolates were from catheterized patients, four (22.22%) were from patients with recurrent UTI, three (16.66%) were from patients with pyelonephritis, and four (22.22%) were from patients with cystitis. This also accounts for 25% of the CAUTI isolates, 36.36% of recurrent UTI isolates, 3.90% of cystitis isolates, and 9.67% of pyelonephritis isolates as MDR. None of the asymptomatic bacteriuria isolates were MDR (**Table 6**). Additionally, among the 180 UPEC, 33 (18.33%) were metallo-beta-lactamase (MBL) producers and 24 (13.33%) were Amp C producers; among these, 18 and 11 isolates were also MDR, respectively.

**Table 6:** Occurrence of MDR-UPEC.

Category of UTI	MDR UPEC (%)	Chi-square	*P*-value
Cystitis (101)	4 (3.90)	85.63	0.001
Pyelonephritis (31)	3 (9.67)	20.16	0.001
Recurrent UTI (11)	4 (36.36)	0.82	0.366
CAUTI (28)	7 (25)	7.00	0.008
ASB (9)	–	–	NA
Total (180)	18 (10%)	115.20	0.001*

Significant P value ≤ 0.05. *Significant association. NA, Not applicable.

### 3.4 Biofilm formation among UPEC and commensal *E. coli*

**Figure 1:**
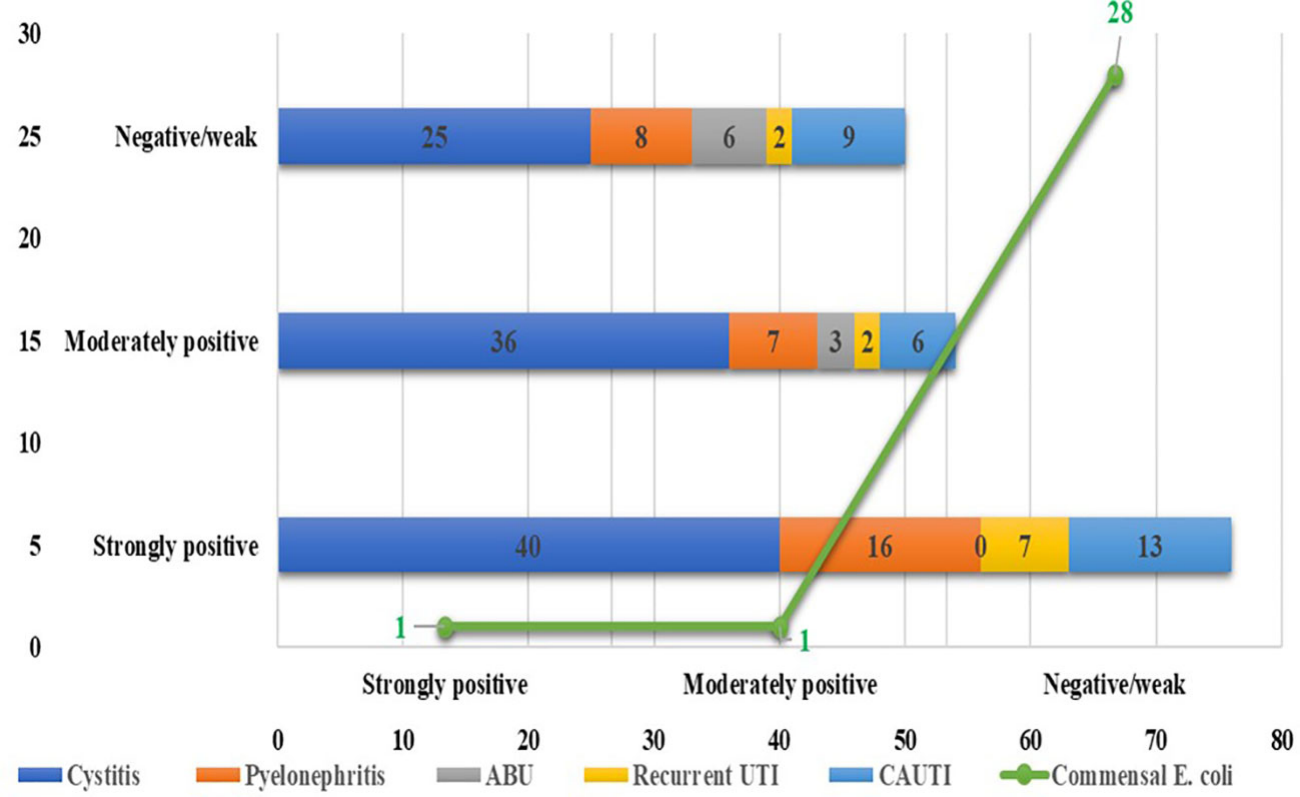
Biofilm detection by Tissue culture plate method.

**Figure 2:**
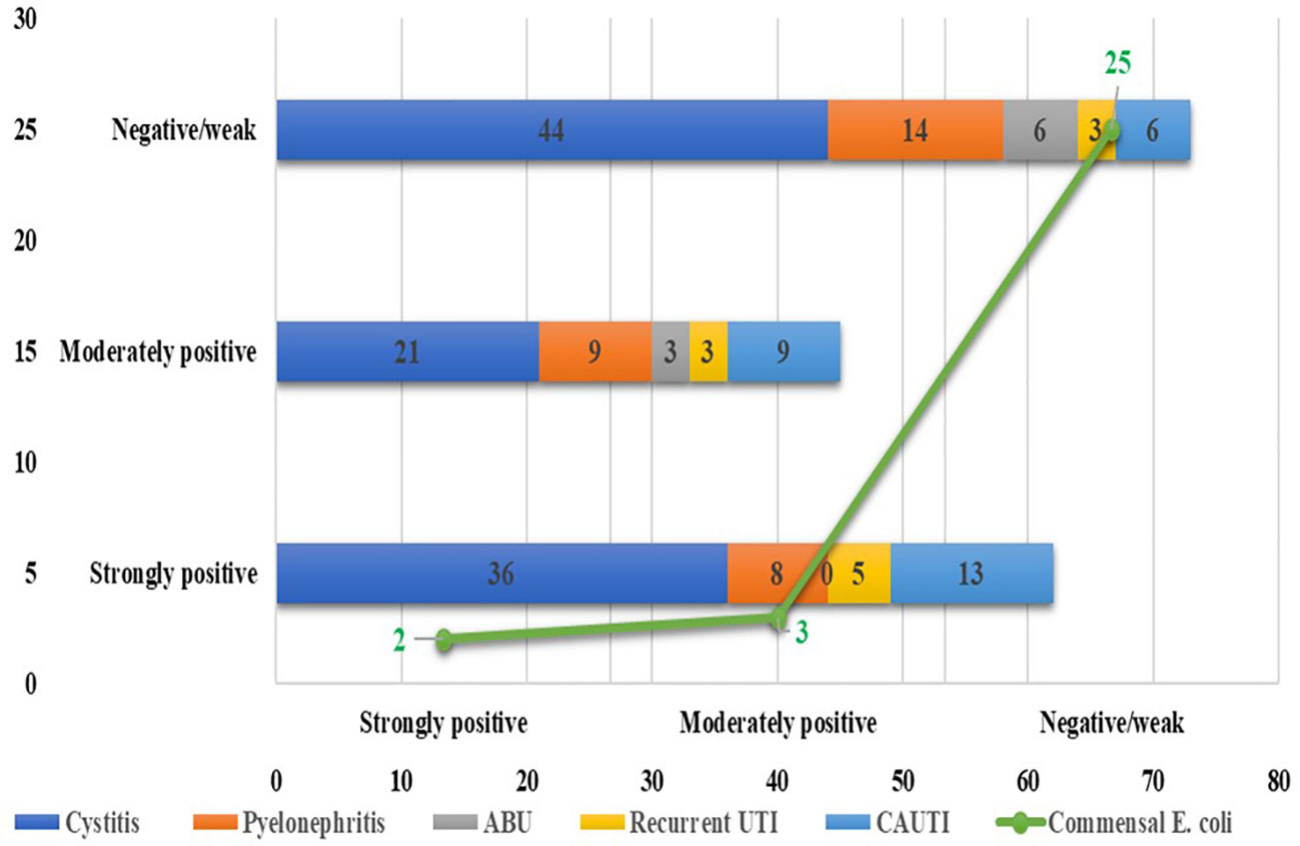
Biofilm detection by the tube adherence method.

**Figure 3:**
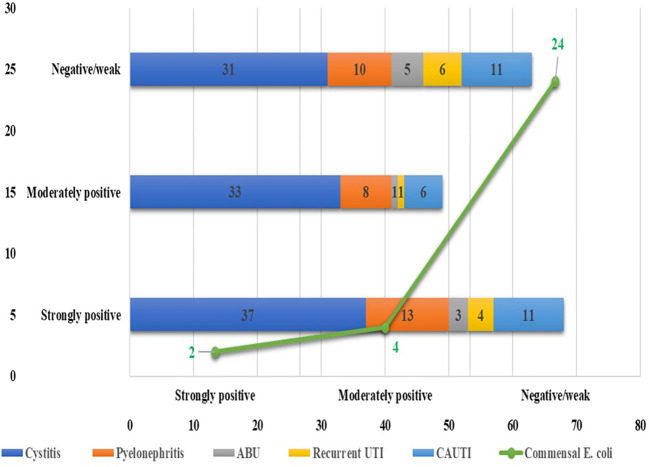
Biofilm detection by Congo-red method.

Among the 180 UPEC isolates, biofilm production was detected in 130 (72.22%) isolates by the tissue culture plate method (**Figure 1**), 107 (59.44%) by the tube adherence method (**Figure 2**), and 117 (65%) by the CRA method (**Figure 3**). In comparison, among the 30 commensal *E. coli* isolates, biofilm formation was observed in only two (6.66%) isolates by the tissue culture plate method, five (16.66%) by the tube adherence method, and six (20%) by the CRA method. The comparison of biofilm detection by different phenotypic methods is provided in **Table 7**.

**Table 7:** Comparison of biofilm detection by different phenotypic methods among UPEC.

Method	Biofilm producers, *n* (%)	Non-biofilm producers, *n* (%)	Comparison to gold standard (TCP)	*P*-value
Congo red agar method	117 (65%)	63 (35%)	TCP vs. Congo red	0.140
Tube adherence method	107 (59.44%)	73 (40.55%)	TCP vs. tube adherence	0.011
Tissue culture plate method	130 (72.22%)	50 (27.22%)	–	

*p* < 0.05, significant difference; *p* ≥ 0.05, not significant.

The tissue culture plate method, considered the gold standard for identifying biofilm producers, identified 130 biofilm producers (72.22%) and 50 non-biofilm producers (27.78%). In comparison, the Congo red agar method identified 117 biofilm producers (65.0%) and 63 non-biofilm producers (35.0%), with no significant difference observed compared to TCP (*p* = 0.140), indicating a comparable performance. However, the tube adherence method identified 107 biofilm producers (59.44%) and 73 non-biofilm producers (40.56%) and showed a significant difference compared to TCP (*p* = 0.011), suggesting that it may underperform relative to the gold standard.

### 3.5 Prevalence of biofilm-forming genes among UPEC and commensal *E. coli*

Real-time multiplex PCR identified the *fimH* gene, which codes for type I fimbriae, in 98.33% of the UPEC isolates. The gene was present in 100% of the pyelonephritis isolates and recurrent UTI and CAUTI isolates, 99% of cystitis isolates, and 77.77% of asymptomatic bacteriuria (ASB) isolates. The *papC* gene was detected in 79 (43.88%) UPEC isolates, with a higher frequency among recurrent UTI, pyelonephritis, and CAUTI isolates. The *sfa/focED* gene, coding for S fimbriae, was found in 21.11% of the UPEC isolates, predominantly in those associated with pyelonephritis and CAUTI. Afimbrial adhesins encoded by *afa/draBC* were observed in only 10.55% of the isolates, mostly among pyelonephritis and cystitis isolates, as detailed in **Table 8**. Additionally, a comparative analysis of the genotypic results of UPEC and commensal *E. coli* is given in **Table 9**.

**Table 8:** Prevalence of biofilm coding genes among various UPEC isolates.

Virulence genes	Positive in cystitis cases (101)	Positive in pyelonephritis cases (31)	Positive in ABU cases (9)	Positive in recurrent UTI cases (11)	Positive in catheterized cases (28)	Total positive cases
*fimH*	100 (99%)	31 (100%)	7 (77.77%)	11 (100%)	28 (100%)	177 (98.33%)
*papC*	4 (3.96%)	8 (25.80%)	0	2 (18.18%)	4 (14.28%)	18 (10%)
*papGII*	11 (10.89%)	19 (61.29%)	1 (11.11%)	6 (54.54%)	14 (50%)	51 (28.33%)
*papGIII*	2 (1.98%)	6 (19.35%)	1 (11.11%)	0	1 (3.57%)	10 (5.55%)
*sfa/focDE*	16 (15.84%)	11 (35.48%)	0	3 (27.27%)	8 (29.28%)	38 (21.11%)
*afa/draBC*	9 (8.91%)	5 (16.12%)	0	1 (9.09%)	4 (4.28%)	19 (10.55%)

**Table 9:** Comparison of the presence of various biofilm genes among UPEC and intestinal commensal *E. coli*.

Virulence genes	Positive in cases (180)	Positive in controls (30)	Chi-square	P-value
*fimH*	177 (98.33%)	20 (66.6%)	44.40	0.001*
*papC*	18 (10%)	3 (10%)	0	1.00
*papGII*	51 (28.33%)	0	11.22	0.001*
*papGIII*	10 (5.55%)	0	1.75	0.186
*sfa/focDE*	38 (21.11%)	0	7.73	0.005*
*afa/draBC*	19 (10.55%)	0	3.48	0.062

*Significant association, significant P value ≤ 0.05.

As shown in **Table 9**, among the 30 intestinal commensal *E. coli* isolates, *fimH* gene was identified in 20 (66.6%) isolates, while the *papC* gene was detected in three (10%) isolates. However, none of the commensal isolates harbored *papGII*, *papGIII*, *sfa/focDE*, and *afa/draBC* genes.

### 3.6 Antimicrobial susceptibility pattern of biofilm-producing and non-biofilm-producing UPEC

Antimicrobial resistance was compared, and it was observed that the biofilm-producing strains were more resistant to the commonly used antimicrobials than the non-biofilm-producing strains. All of the biofilm-forming isolates were sensitive to fosfomycin and tigecycline, as shown in **Table 10**.

**Table 10:** Antimicrobial susceptibility pattern of biofilm-producing and non-biofilm-producing UPEC.

Antimicrobial agent	Biofilm producers (130)		Non-biofilm producers (50)		Chi-square	*P*-value
	Sensitivity	Resistance	Sensitivity	Resistance		
Fosfomycin (E-test)	130 (100%)	0 (0%)	0 (0%)	–	NA	NA
Tigecycline	130 (100%)	0 (0%)	30 (100%)	(0%)	NA	NA
Nitrofurantoin	114 (87.7%)	16 (12.30%)	47 (90%)	3 (10%)	1.52	.217
Amikacin	107 (82.31%)	23 (17.69%)	48 (96%)	2 (4%)	5.66	.017
Gentamycin	100 (76.93%)	30 (23.07%)	43 (86%)	7 (14)	1.82	0.177
Imipenem	83 (63.85%)	47 (36.15%)	38 (76%0	12 (24%)	2.42	0.120
Meropenem	79 (60.77%)	51 (39.23%)	35 (70%)	15 (30%)	1.32	0.250
Ciprofloxacin	68 (52.31%)	62 (47.69%)	48 (96%)	2 (4%)	30.09	.001*
Cefaperazone/sulbactam	75 (57.70%)	55 (42.30%)	26 (52%)	24 (48%)	0.48	.491
Nalidixic acid	54 (41.54%)	76 (58.46%)	38 (76%)	12 (24%)	17.16	.001*
Cefepime	31 (23.85%)	99 (76.15%)	28 (56%)	22 (44%)	15.26	.001*
Ceftriaxone	26 (20%)	104 (80%)	23 (46%)	27 (54%)	12.32	.001*
Cefuroxime	18 (13.85%)	112 (86.15%)	26 (52%)	24 (48%)	28.46	.001*
Amoxiclav	19 (14.62%)	111 (85.38%)	13 (26%)	37 (74%)	3.20	.074
Ampicillin	8 (6.16%)	122 (93.84%)	6 (12%)	44 (88%)	1.72	.190

*Significant P value ≤0.05. *Significant association. NA, not applicable.

As shown in **Table 11**, 18 of the 180 UPEC isolates were MDR, of which 13 were biofilm-forming, accounting for 72.22% (13/18) of the MDR isolates to be biofilm-forming. Of the 130 biofilm-forming UPEC, 13 (10%) were MDR isolates, 33.33% of the biofilm-forming recurrent UTI isolates were MDR, and 26.31% of the biofilm-forming catheter isolates were MDR. Of the 130 biofilm-forming isolates, 25 (19.23%) were MBL producers, and among these 25, 13 were also MDR isolates. Among 130 biofilms producing UPEC, 17 (13.07%) were AmpC producers, and among the 17, 10 were MDR isolates.

**Table 11:** Biofilm-forming MDR UPEC isolates in various categories of UTI.

Category of UTI	Biofilm-forming MDR UPEC (%)	*P*-value
Cystitis (76)	4 (5.26)	0.001*
Pyelonephritis (23)	1 (4.34)	0.001*
Recurrent UTI (9)	3 (33.33)	0.317
CAUTI (19)	5 (26.31)	0.039
ASB (3)	–	NA
Total (130)	13 (10)	0.001*

Significant *P* value ≤ 0.05. *Significant association. NA, not applicable.

## 4 Discussion

UTIs are among the most common bacterial infections in humans. UTIs are a common cause of morbidity and affect persons of all age groups, including young women, children, and the elderly. *E. coli* accounts for more than 80%–90% of all UTIs (Desai et al., 2013). The ability of UPEC to cause UTI is associated with the expression of a variety of virulence factors (Klemm and Schembri, 2000; Roos et al., 2006). The severity of the infection is dependent on both the virulence of UPEC and also on the susceptibility of the host (Santo et al., 2006). One of the important virulence factors of UPEC is biofilm formation; understanding the rate of biofilm formation will aid in the proper management and initiation of appropriate antibiotics, which will help in the prevention of antimicrobial resistance.

It was observed in our study that among the 180 *E. coli* isolates, 101 (56.11%) were from patients with cystitis, 31 (17.22%) were from patients with pyelonephritis, 28 (15.55%) were from catheterized patients, 11 (6.11%) were from patients with recurrent UTI, and nine (5%) were from patients with asymptomatic bacteriuria. The main treatment modality for UTIs is the use of antibiotics such as β-lactams, trimethoprim, nitrofurantoin, and quinolone, but due to the widespread misuse of these antibiotics, strains of the isolates have developed, thus making the sensitivity reports essential for the selection of appropriate antibiotics. In this study, it is observed that UPEC exhibit a high degree of resistance to commonly used antibiotics like ampicillin, cefepime, cefuroxime, and ceftriaxone. A maximum number of isolates were sensitive to nitrofurantoin, amikacin, and carbapenems. Furthermore, 100% sensitivity was observed from tigecycline and fosfomycin; these observations correlated with the observations made by Manjula A Vagarali et al. (Annapurna et al., 2014a), M. Eshwarappa et al. (Eshwarappa et al., 2011), and Arindam Chakraborty et al. (Chakraborty et al., 2017). The antibiotic resistance pattern of UPEC was compared with fecal *E. coli* isolated from healthy individuals. We found that fecal *E. coli* was more sensitive to the antibiotics tested than the UPEC isolates.

UPEC is one of the most common uropathogens associated with UTI (Raksha et al., 2003; Davis and Flood, 2011; Foxman, 2014). To initiate an infective process in the urinary tract, UPEC has to survive the host defense mechanisms like exfoliation of uroepithelial cells, micturition, and endogenous antimicrobial agents. Given this, UPEC possesses many virulence and fitness factors (Dhakal et al., 2008). Genes located on the pathogenicity island code for the various VFs in UPEC (Rao, 1994). The severity of UTI depends on the number of virulence factors expressed by the uropathogen and also on host susceptibility (Fowler and Stamey, 1977).

In our study, biofilm production was observed in 72.22% of UPEC and was found to be one of the most common virulence factors among UPEC. A similar observation was made by Lalith Meshram et al. (Mesharam et al., 2012), who reported that 76% of UPEC have biofilm-forming capacity. Shah et al. (2019) reported in their study that 62% of UPEC were biofilm producers, and Annapurna et al. (2014b) have reported 73.30% of the isolates to be biofilm producers (Annapurna et al., 2014a). E. Suman et al. (Suman et al., 2007), in their study, also reported a very high rate (92%) of biofilm-forming UPEC, and our results are in contrast to the results obtained by Pramodhini and Niveditha (2012) who reported only 39.60% of *E. coli* isolates to be biofilm producers. Biofilm formation is an important virulence factor which gives several survival advantages to the isolate. It allows them to persist in the urinary tract by protecting them from host defense mechanisms, and it also renders the isolates resistant to antimicrobial agents, which interferes with their eradication (Jadhav et al., 2011; Fattahi et al., 2015; Vijayalakshmi et al., 2015).

In this study, 6.6% of the commensal *E. coli* were biofilm producers; this finding is statistically significant. A similar observation was made by Fattahi et al., Karam et al., and Soto et al., who have reported a higher prevalence of biofilm formation among UPEC than in controls (Jadhav et al., 2011; Fattahi et al., 2015; Vijayalakshmi et al., 2015; Karam et al., 2018). However, 6% of our control strains were biofilm producers. This could be because it is the usual living condition of bacteria in natural environments. Biofilm formation was most commonly associated with UPEC isolated from CAUTI (92.85%), followed by isolates from recurrent UTI (81.81%) and acute cystitis, and only 11.1% of ABU isolates were biofilm producers. These findings are in agreement with the findings of Karigoudar et al. (2019) who have reported 89.5% of CAUTI isolates to be biofilm producers. Tabasi et al. (2015) have reported a high incidence of biofilm production among recurrent UTI isolates, which is in correlation with our study.

In the present study, biofilm-forming UPEC were resistant to ampicillin (93.84%), followed by amoxiclav (85.38%), cefuroxime (86.15%), ceftriaxone (80%), and cefepime (76.15%). Most of the biofilm-producing UPEC were sensitive to aminoglycosides and carbapenems, and all of the strains were sensitive to nitrofurantoin, tigecycline, and fosfomycin. A similar high resistance to various antibiotics was noted by Tajbakhsh et al., R. karigowder et al., Poovendran et al., Karam et al., Tadepalli et al., Tabasi et al., and Sevanan et al., all of whom have also reported a higher frequency of resistance to antibiotics among biofilm producers (Sevanan et al., 2011; Poovendran and Ramanathan, 2014; Tabasi et al., 2015; Tadepalli et al., 2016; Tajbakhsh et al., 2016; Karam et al., 2018; Karigoudar et al., 2019). In the present study, a comparative increase in the resistance among biofilm producers to all the tested antibiotics was noted; however, a statistically significant correlation was observed with nalidixic acid, ciprofloxacin, cefuroxime, ceftriaxone, and cefepime.

In our study, 18 out of the 180 UPEC were found to be MDR, of which 13 were biofilm producers; this accounted for 72.22% (13/18) of the MDR isolates to be biofilm producers. Of the 130 biofilms forming UPEC, 13 (10%) were MDR isolates. In addition, 33.33% and 26.31% of the biofilm-forming UPEC from recurrent UTI and CAUTI were MDR. A statistically significant association between biofilm production and MDR was observed. This is in contrast to the study by Shrestha et al. (Shrestha et al., 2019). The higher rate of resistance among biofilm producers is attributed to insufficient antimicrobial concentration within the biofilm matrix, delayed penetration of the antibiotic into the deeper layers of the biofilms, and the relatively inactive state of the isolate within the biofilm (Fattahi et al., 2015). We report a higher rate of MDR UPEC, and most of the MDR isolates were from CAUTI. These isolates were MDR as they were nosocomial strains and, secondly, because of selective pressure due to the over-the-counter use of broad-spectrum antibiotics.

It is well known that one of the characteristic features of biofilm is its ability to tolerate antibiotics (Bjarnsholt et al., 2005). It is reported that bacteria in biofilms tolerate 100–1,000 times higher concentrations of antibiotics than planktonic cells (Mah and O’Toole, 2001; Donlan and Costerton, 2002; Alhede et al., 2011). Thus, there may be treatment failure when the choice of antibiotics is done based on the MIC report, as MIC determines the minimum inhibitory concentration against the planktonic state and does not quantify the concentration required to inhibit bacteria in biofilm. Thus, determination of MBEC will be more useful to adjust the dosage of the antibiotic required to eradicate bacteria in the biofilm mode of life (Ghanwate, 2012). The MBEC assay was developed by Ceri et al. (Sepandj et al., 2004). The MBEC assay was done to evaluate the change in the susceptibility pattern of one of the common antibiotics used to treat UTI—ciprofloxacin—and one less commonly used antibiotic in our setting—fosfomycin.

In this study, genes coding for adhesins like *fimH*, *papC*, *papGII*, *papGIII*, *Sfa/focDE*, and *afa/draBC* were studied. The *fimH* gene codes for type I fimbriae, and it was present in 98.33% of the UPEC isolates. The gene was present in 100% of the pyelonephritis, recurrent UTI, and CAUTI isolates and was found in 99% of the cystitis isolates and 77.77% of the ABU isolates. The fimH gene was seen in 98.33% of UPEC and 66/6% of fecal *E. coli*. This result is comparable to the studies conducted by Arindam Chakraborty et al. (189) (90%), Kudinha et al., and Mora et al., who have also demonstrated a high prevalence of *fimH* genes among the UPEC isolates (Martinez et al., 2000; Mora et al., 2009; Chakraborty et al., 2017). Yun et al. (2014) have reported *fimH* to be present in 100% of pyelonephritis isolates and 96% of ABU isolates, which is under our study. Type 1 fimbria was commonly associated with cystitis and was found to help in the development of IBCs in a mouse model of UTI (Connell et al., 1996; Johnson et al., 2001; Gunther NW et al., 2002). Type 1 fimbriae facilitate bacteria to adhere to each other, resulting in biofilm-like communities in the urinary tract.

P fimbriae, the principal mannose-resistant adherence organelle of UPEC, contributes to pathogenesis as it is involved in bacterial colonization and stimulates an injurious host inflammatory response (Tiba et al., 2008). Within the Pap operon, there are genes which code for the outer membrane protein (papC), a minor structural subunit of the fimbriae (*papE/F*) and *papG* adhesins (*papGI/papGII/papGIII*) (Daigle et al., 1994). The Pap G II adhesin is associated with the strains causing pyelonephritis and bacteremia, while the Pap G III adhesin is associated with the strains causing cystitis (Féria et al., 2001; Ghazvini et al., 2019), and PapGII adhesin is prevalent among fecal isolates.

In the present study, the *pap* gene was present in 79 (43.88%) of the UPEC isolates. Similar observations were made by Arindam Chakraborty et al. who reported 49% and Ki Wook Yun et al. who reported 45.3% of their isolates to be positive for the pap gene (Yun et al., 2014). In our study, it was observed that the occurrence of various *Pap* genes (*papC*, *papGI*, and *papGII* was 25%, 51%, and 19% respectively) was more frequent in pyelonephritis strains, which is in concordance with the studies conducted by Ghazvini et al. (36%). Mabbett et al. have reported a slightly higher frequency of this gene among pyelonephritis strains than in our study (Lane and Mobley, 2007; Mabbett et al., 2009). In the present study, it was also found that the *papGII* gene occurred more frequently in pyelonephritis isolates. This is in agreement with the findings of Monique et al. However, *papGIII* was not commonly associated with cystitis strains in our study (Daigle et al., 1994).

P fimbriae are most commonly expressed by isolates causing pyelonephritis, recurrent UTI, and CAUTI (Herias et al., 1995); however, these fimbriae are also required by the commensal *E. coli* for colonization and persistence in the gut. At times, these fimbriae help fecal *E. coli* to spread to extra-intestinal sites like the urinary tract (Wold et al., 1992; Malagolini et al., 2000). Moreover, 10% of the control strains in this study harbored the *papC* gene. The *sfa/foc ED* gene codes for S fimbriae, which recognizes and adheres to the sialic acid expressed on the receptors of epithelial cells in the kidney and vascular endothelial cells. Sialic acid residues are also found in uroplakin proteins present on the bladder luminal surface and, thus, may have a role in the pathogenesis of cystitis (Wold et al., 1992).

In our study, *Sfa/foc ED* was seen in 21.11% of the UPEC isolates and was more commonly associated with pyelonephritis and CAUTI isolates. This finding is in concordance with the findings of Monique et al. (27.8%) (Malagolini et al., 2000). Ki Wook Yun et al. (Goluszko et al., 1997 have reported a slightly lower prevalence of this gene (15.6%). The *afa/Dr* family consists of both fimbrial adhesive organelles and the afimbrial adhesins. The *afa/Dr*-associated proteins are invasins, which help in the internalization of bacteria by the host cells (Behzadi, 2017). Afimbrial adhesins coded by *afa/draBC* were seen in only 10.55% of the isolates and were more commonly observed in pyelonephritis and cystitis isolates. Shahin et al. have also reported a low prevalence of this gene among UPEC. However, the literature review has reported that up to 65% of UPEC cause cystitis, 26% cause pyelonephritis, and 6% cause asymptomatic bacteriuria to harbor this gene (Wood, 2009). None of the control strains harbored this gene.

*Limitations of the study*: The molecular detection of *AmpC* and MBL could not be done because of financial constraints, and antibiotic susceptibility testing was interpreted using guidelines representing the serum concentration of the antibiotics.

## 5 Conclusion

*E. coli* is a major pathogen causing UTIs. Many VFs in UPEC have been studied, and it is observed that no single VF could be established as a marker of urovirulence, thus suggesting the role of multiple virulence factors in uropathogenesis. However, biofilm formation is consistently seen in recurrent UTI, CAUTI, and pyelonephritis cases and thus could be used as a marker for such complications, thereby helping in choosing the appropriate dosage for treatment. A genotypic study helps in understanding the molecular pathogenesis, which, in turn, will help in developing clinical strategies for the prevention and management of UTIs. It was observed that the majority of the UPEC were biofilm-forming, and there was a significant difference in the antibiotic susceptibility pattern of biofilm producers and non-biofilm producers. Treatment of UTI is by selecting an antibiotic that the isolate is sensitive to, but the sensitivity reports are generated against planktonic bacteria and may not be effective against the organisms within the biofilms, resulting in recurrent infections and treatment failures. Thus, to conclude, it is important to incorporate routine biofilm detection and MBEC detection methods in a diagnostic laboratory, which will help in the appropriate selection of the dose of the antibiotic, which, in turn, will help overcome treatment failures.

## Data Availability

The original contributions presented in the study are included in the article/supplementary material. Further inquiries can be directed to the corresponding author.
